# Rapid method for plutonium-241 determination in soil samples

**DOI:** 10.1007/s10967-013-2907-y

**Published:** 2014-01-24

**Authors:** M. Piekarz, A. Komosa

**Affiliations:** 1 Central Laboratory for Radiological Protection, Konwaliowa 7, 03-194 Warsaw, Poland; 2 Maria Curie-Sklodowska University, Pl. M. C. Sklodowskiej 3, 20-031 Lublin, Poland

**Keywords:** Plutonium, ^241^Pu, Liquid extraction, Liquid scintillation spectrometry, Alpha spectrometry, Soil samples

## Abstract

A simple and rapid procedure for the determination of plutonium isotopes in the environment is presented. The procedure combines alpha spectrometry, solvent extraction and liquid scintillation measurements to ensure that both alpha- and beta-emitting isotopes are determined. Of five tested extractants, bis-(2-ethylhexyl) phosphoric acid was found to be the best choice. The procedure was applied to soil samples contaminated with Chernobyl fallout.

## Introduction

Alpha spectrometry is the most widely used method for the determination of plutonium isotopes in environmental samples. However, this method does not permit the determination of beta-emitting ^241^Pu, a radionuclide abundant in the environment. This isotope decays to ^241^Am, another radiotoxic hazard [1]. The best way to determine ^241^Pu in an environmental sample is by liquid scintillation (LS) after the isotope has been removed from the sample by a radiochemical procedure. The following procedure can be used for the simultaneous determination of alpha and beta plutonium activity [2–4]. An important consideration is choosing an extracting agent that is both efficient and minimizes quenching effects during LS measurements.

The aim of this study is to illustrate a rapid method for the determination of ^241^Pu in soil samples using a simple solvent extraction method followed by LS measurements. The procedure described here is applied after the determination of other Pu isotopes in the sample by a technique of alpha spectrometry.

## Experimental

There are many commonly known extracting agents for the extraction of heavy metal traces, such as: trioctylphosphine oxide, TOPO [5–7], bis-(2-ethylhexyl) phosphoric acid, HDEHP [8, 9], methyltrioctylammonium chloride (Aliquat 336) [10, 11], *N,N*-diethyldodecanamide, DEDA [12] or thenoyltrifluoracetone, HTTA [13]. These compounds have been tested to find those of high plutonium recovery from the environmental samples. This test should precede the final LS measurements.

The ultra low-level spectrometer Quantulus 1220 (Wallac, Perkin-Elmer) was used for LS measurements. Permablend III (Packard), consisted of 91 % PPO and 9 % bis-MSB dissolved in toluene was applied as a scintillation cocktail. Samples were measured for 5 h, after 24 h stabilization in darkness in the apparatus to avoid chemiluminescence. Standard solutions of ^242^Pu (~0.02 Bq) in 3 M nitric acid were used as tracers.

Alpha spectrometric measurements were performed using the alpha spectrometer (model 7401 Canberra) with PIPS detector of 17 keV FWHM resolution, the 1520 mixer-router, S-100 multichannel analyzer, and Genie™ 2000 software for quantitative analysis (Canberra).

Before the LS measurements, the experiments were performed to choose a proper extractant which is characterized by the best efficiency and low quenching effects. The following extractant solutions were tested: 0.2 M HDEHP dissolved in toluene, 0.2 M TOPO in cyclohexane, 0.05 M Aliquat 336 in xylene, 0.3 M DEDA in toluene and 0.01 M HTTA in toluene. The extraction procedure was described previously [14, 15].

For the verification of the procedure, soil samples contaminated with Chernobyl fallout were analyzed. The samples were collected in 1991 near Bragin village from two successive layers of soil (0–5 and 5–10 cm). The samples were ashed, co-precipitated, separated by ion exchange and then electrodeposited on small steel plates, as described recently [16]. After the alpha-spectrometry measurements, the electro-deposited plutonium was washed off of the steel plates with nitric acid and extracted into the organic phase of 0.2 M HDEHP solution in toluene. An aliquot of the organic phase was mixed with the scintillation cocktail and its activity was measured with a Quantulus spectrometer.

## Results and discussion

The characteristics of a useful extractant include the following: acceptable counting efficiency, resistance to decomposition by nitric acid, miscibility with the scintillation cocktail, low background counts, and low quenching characteristics. HDEHP had the best combination of these characteristics.

**Fig. 1:**
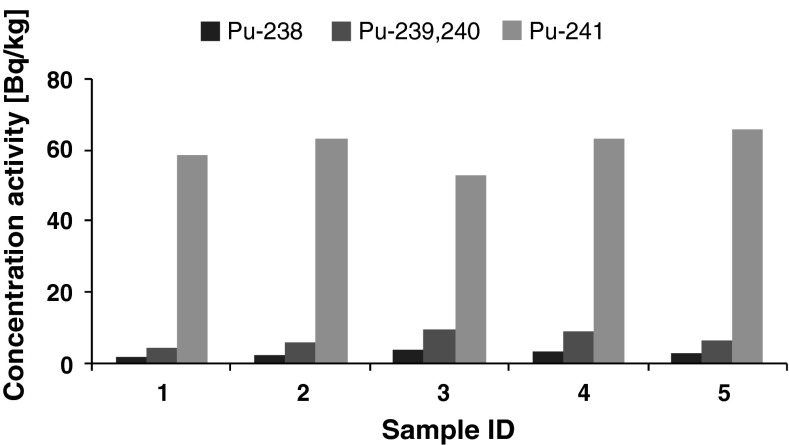
Activity concentration of plutonium (Bq/kg) in soil layer 0–5 cm (five sample replicates)

**Fig. 2:**
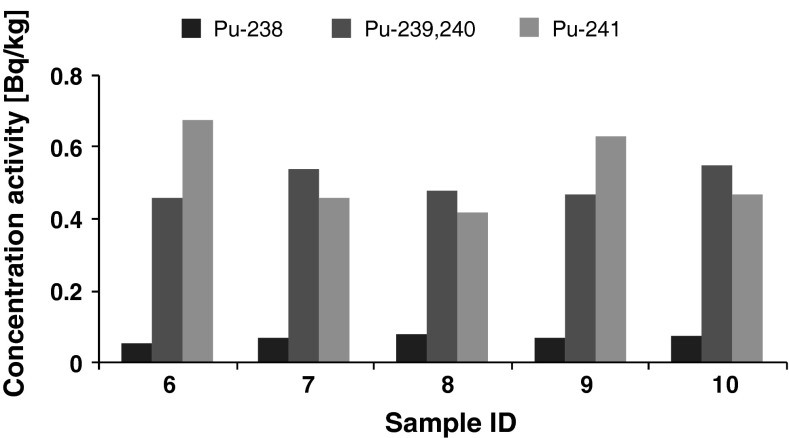
Activity concentration of plutonium (Bq/kg) in soil layer 5–10 cm (five sample replicates)

The activities of ten soil samples are shown in Figs. 1, 2. They were collected and prepared as described in [16, 17]. Figure 1 illustrates the activities of five random soil samples taken from a soil depth 0–5 cm deep. Figure 2 illustrates another set of five samples taken from 5 to 10 cm soil depth.

Figure 1 illustrates that the surface layer of soil (0–5 cm deep) displayed the following activities: ^238^Pu of 2.88 Bq/kg, 6.98 Bq/kg of ^239+240^Pu and 60.8 Bq/kg of ^241^Pu. Figure 2 illustrates that the deeper layer of soil (5–10 cm deep) displayed the following activities: 0.066 Bq/kg of ^238^Pu, 0.50 Bq/kg of ^239+240^Pu and 0.53 Bq/kg of ^241^Pu. The estimated uncertainties of these results are about 10 % for both soil depths.

Calculation of the isotopic ratios: ^238^Pu/^239+240^Pu and ^241^Pu/^239+240^Pu allows the estimation of the plutonium origin. Calculated isotope ratios are presented in Table 1. They can be interpreted in a historical context: what is the origin of the plutonium in the soil samples? Global fallout plutonium reveals a very small share of ^238^Pu (about 4 %). On the contrary, the Chernobyl fallout includes almost 50 % of ^238^Pu [17, 18].

**Table 1:** Isotopic ratios of plutonium in analyzed Chernobyl contaminated samples (the last column ratio has been recalculated back on the date of the Chernobyl accident)

Layer	^238^Pu/^239,240^Pu	^241^Pu/^239,240^Pu	^241^Pu/^239,240^Pu in 1986
0–5 cm	0.42	9.5	32
5–10 cm	0.13	1.1	3.6

Table 1 shows the calculated isotopic ratios of the two soil layers. According to the literature data, the typical global fallout ratio ^238^Pu/^239+240^Pu is ranged 0.03–0.05, and for Chernobyl fallout is 0.3–0.65 [19]. The observed ratio indicates that the upper soil layer is contaminated mainly with Chernobyl fallout and the lower rather with a global one.

The data from the literature is as follows, the ratio ^241^Pu/^239+240^Pu for global fallout is about 4.2 (based on the year 1986), and for the Chernobyl fallout it is about 94.8 and varies widely [20]. As seen in Table 1, the calculated average ratio ^241^Pu/^239+240^Pu in the surface layer is 9.5 and after calculation on the date of the Chernobyl disaster it will be equal to 32. In the deeper layer the ^241^Pu/^239+240^Pu ratio was 1.1. It was therefore about 3.6 in 1986. Comparing these results with the literature data, one can conclude that in the deeper soil layer plutonium from global fallout predominates and in the surface layer the contribution of plutonium from Chernobyl fallout is undoubtedly the largest.

## Conclusions

The presented procedure which combines the alpha spectrometry and the LS measurements after solvent extraction may be useful for the rapid determination of all plutonium isotopes present in the environment (^238^Pu, ^239+240^Pu and ^241^Pu).

It was found that among five various extractants, the HDEHP reveals the best properties to be applied as an extracting agent during the sample preparation to LS measurement.

The elaborated method used for the determination of plutonium in the samples contaminated with Chernobyl fallout shows its usefulness. Calculated isotopic ratios confirmed a large share of this source of contamination in the analyzed samples, especially in those taken from the upper layer of soil.
